# Advances in Microflow Cytometry-Based Molecular Detection Methods for Improved Future MDS Cancer Diagnosis

**DOI:** 10.3390/cimb46080476

**Published:** 2024-07-26

**Authors:** Marc Gonsalves, Andres Escobar, Ahmad Diaa Altarabishi, Chang-Qing Xu

**Affiliations:** 1 Department of Engineering Physics, McMaster University, 1280 Main Street West, Hamilton, ON L8S 4L8, Canada; gonsam2@mcmaster.ca; 2 Faculty of Health Sciences, McMaster University, 1280 Main Street West, Hamilton, ON L8S 4L8, Canada; 3 School of Biomedical Engineering, McMaster University, 1280 Main Street West, Hamilton, ON L8S 4L8, Canada; escoba3@mcmaster.ca (A.E.); altaraba@mcmaster.ca (A.D.A.)

**Keywords:** microfluidics, diagnostics, MDS, flow cytometry, microflow cytometry

## Abstract

Myelodysplastic syndromes (MDS) are a rare form of early-stage blood cancer that typically leads to leukemia and other deadly complications. The typical diagnosis for MDS involves a mixture of blood tests, a bone marrow biopsy, and genetic analysis. Flow cytometry has commonly been used to analyze these types of samples, yet there still seems to be room for advancement in several areas, such as the limit of detection, turnaround time, and cost. This paper explores recent advancements in microflow cytometry technology and how it may be used to supplement conventional methods of diagnosing blood cancers, such as MDS and leukemia, through flow cytometry. Microflow cytometry, a more recent adaptation of the well-researched and conventional flow cytometry techniques, integrated with microfluidics, demonstrates significant potential in addressing many of the shortcomings flow cytometry faces when diagnosing a blood-related disease such as MDS. The benefits that this platform brings, such as portability, processing speed, and operating cost, exemplify the importance of exploring microflow cytometry as a point-of-care (POC) diagnostic device for MDS and other forms of blood cancer.

## 1. Introduction

Cancer remains one of the most prolific and widespread causes of death across the globe. The World Health Organization (WHO) estimated the number of new cancer cases in 2022 to be 20 million people worldwide, while expecting the number to rise to a staggering 35 million by 2050—a 77% increase [1]. Early detection and treatment of cancers has been an effective tool in successfully treating cancers into remission. However, this process is plagued by limitations in diagnostic technique specificity, sensitivity, and turnaround time, as well as patient safety concerns. Historically, commonplace diagnostic techniques such as low dose computed tomography (LDCT), mammography, magnetic resonance imaging (MRI), and sputum cytology were used to try and detect and diagnose cancers in conjunction with one another. However, due to some of the inherent limitations associated with these diagnostic methods, such as cost, specificity, and detection limits, their diagnostic potential was left somewhat wanting [2,3]. Over time, to try and address these limitations, a more recent technological advancement known as flow cytometry was being explored. By providing a rapid throughput platform for real-time cell analysis, flow cytometry is not only able to rapidly identify cells in a more rapid and accessible manner than a lot of conventional techniques, but it also reduces the workload of healthcare professionals and provides alternatives to exposing patients to risks such as radiation.

**Figure 1:**
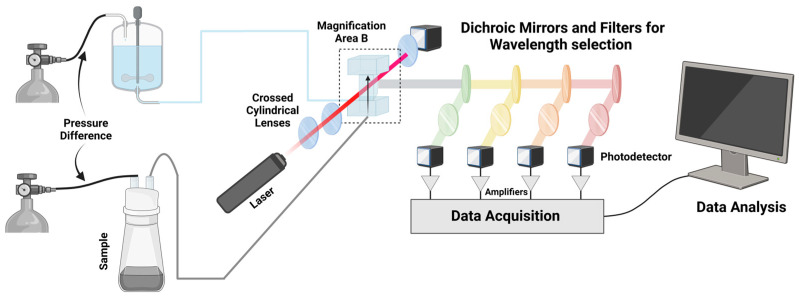
Example of flow cytometry set-up, adapted from [4].

Conventional flow cytometry involves the delivery of sheath fluid and a sample to a flow chamber with pumps or pressure in the tank or container [4] (Figure 1). The sheath fluid is pumped at a high volumetric rate relative to the sample, which allows the sample to be physically focused before entering the flow cell. Once the sample has entered the flow cell in the form of an approximately 5 μm diameter stream, a light source such as a laser is emitted and focused through beam-shaping optics in order to drive it towards the flow cell. This then forms an interrogation volume, which is essentially the volume of the sample stream exposed to the laser. The light emitted orthogonally to the incident laser is then split through dichromic mirrors, ultimately being brought to photodetectors such as photomultiplier tubes and collected for analysis [5]. Parameters such as forward scatter (FSC, light scattered at a small angle from the axis of the input beam) and side scatter (SSC) demonstrate the size of the cell and its granularity, respectively [6]. It is possible to gain insight on 19 optical parameters for a cell through multiple interrogation points, lasers, and optical collection paths [7].

In recent years, flow cytometers have demonstrated their ability to more accurately and readily detect biomarkers associated with the presence of cancers in the human body for a more effective form of diagnosis. This increased diagnostic potential has made flow cytometry the current gold standard in cancer biomarker detection and analysis. Biomarkers such as prostate-specific antigen (PSA), cancer-antigen 125 (CA-125), and carcinoembryonic antigen (CEA) [8] are commonly sought-after detection targets that allow healthcare specialists to loosely attribute the upregulation of these biomarkers to the presence of cancers in the body. Once screened, however, determining the specific type of cancer can be an issue as many of these biomarkers overlap between cancers. In the case of myelodysplastic syndrome (MDS), its overlap of biomarkers with other more common forms of cancer [9] is especially troubling given the high lethality rate of MDS [10].

MDS is a rare form of blood cancer that is known to rapidly progress into malignant leukemia, causing death in many cases. The biomarkers associated with this are not very well defined and often overlap with several other forms of cancer, making diagnosis extremely difficult. Moreover, MDS diagnoses often require a combination of clinical history, peripheral blood tests, and bone marrow cell morphology data to achieve a sufficiently accurate diagnosis [11]. In the hopes of addressing these limitations, 18 European countries took part in establishing a standardized approach for the use of flow cytometry in specifically diagnosing MDS during a summit in 2008 [12]. The result ended with a focus on immunophenotyping. Immunophenotyping in flow cytometry can be used to identify and quantify irregularities in cancer-linked cells, biomarkers, and gene expression, which ultimately lead to a diagnostic conclusion. Many of the most recent examples of flow cytometry-based immunophenotyping detection focus on observing the maturation patterns of granulocytes, monocytes, and lymphocytes to produce a flow cytometry scoring method that is used to produce a diagnosis or prognosis [13]. Some of the most commonly used flow cytometry scoring methods for the diagnosis of MDS and the prediction of outcomes are the Ogata score, the International Prognostic Scoring System (IPSS), and the Revised International Prognostic Scoring System (IPSS-R). To further enhance MDS detection, novel scoring systems such as the Molecular International Prognostic Scoring System (IPSS-M) have also been proposed. Nearly all of these scoring systems consider abnormalities in the number of bone marrow blasts, cytogenic factors, and the degree of cytopenia that a patient has achieved at the time of diagnosis and have a varying range of specificity and sensitivity, as discussed in Table 1.

While flow cytometry does provide various improved diagnostic advantages over previously explored diagnostic techniques, there are still several areas in which it can be further improved upon. Commercial flow cytometers tend to be bulky and thus impractical for point-of-care (POC) usage [6]. In addition to their physical limitations, flow cytometers are often challenged with complicated processing, significant reoccurring maintenance costs, and require a skilled operator for usage, which also limit their practicality in analyzing a small volume sample effectively. As a result, to better overcome these lingering limitations, the integration of microfluidic technology with flow cytometry has more recently demonstrated its potential as a potential evolution of the current diagnostic gold standard.

Microfluidic-integrated flow cytometry, otherwise known as microflow cytometry, combines several different micro-fabrication techniques, such as UV lithography and micropattern stamping, with the design principles of a conventional flow cytometer to create microfluidic devices capable of high-throughput, rapid, sample analysis. Ateya et al. described the microfluidic approach to creating a flow cytometer as four key points [21]:

1. focusing the particles to be analyzed in the microfluidic channel,
2. miniaturization of the fluid-handling components,
3. miniaturization of the optics, and
4. integration and applications development.

**Figure 2:**
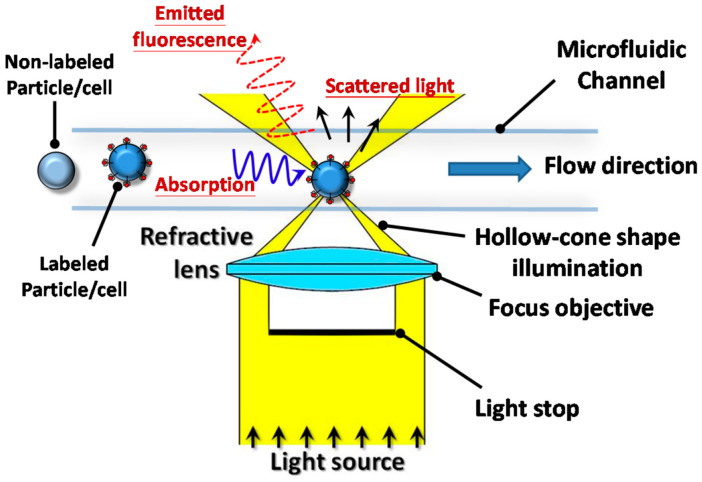
Microfluidic concept, reprinted from Lin et al. [23].

These devices rely on an integrated system that uses a microchip device for the management of flow, known as a microfluidic device. Microfluidic devices are capable of analyzing cells, bacteria, molecular biology, and cellular DNA through sorting within a fluid sample and utilizing fluorescent markers such as antibodies (Figure 2) [22].

**Figure 3:**
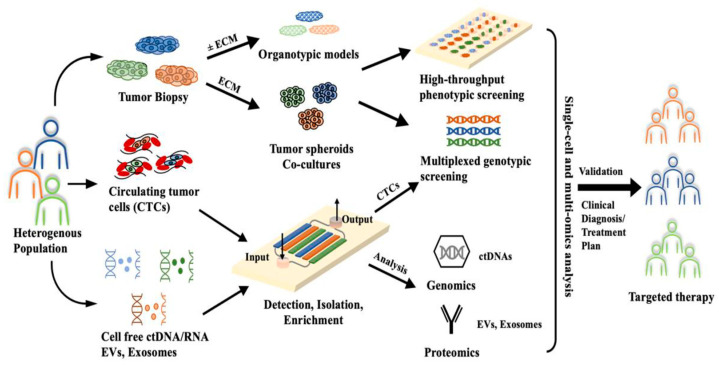
Proposed microfluidic application to a cancer treatment plan, reprinted from Pillai et al. [24].

Recent proposals by individuals such as Pillai et al. demonstrate potential workflows for the diagnosis of cancer through the utilization of microfluidic devices (Figure 3) [24] and have recently started being developed for the diagnosis of blood-related cancers such as leukemia [25]. In contrast to larger-scale devices like most flow cytometers, continuous microfluidic devices utilize laminar flow, which allows for streams of particles to be not only continuous but also more purified as a result of proper separation and a lack of mixing between streams [26]. One interesting avenue of microfluidics is controlled droplet microfluidics, which are capable of using micro-scale laminar flow for individual droplets for individual cell analysis, a feature not common among diagnostic platforms [27,28]. Droplet microfluidic systems segregate these molecules through individual droplet microenvironments, which stem from controlling the flow of a transporter fluid across one or more sample fluids at a junction [28]. These microfluidic devices have shown great promise in identifying bloodborne biomarkers, and are thus potentially relevant to the diagnosis of MDS at a clinical level.

This paper will review recent advancements in microflow cytometry and explore how they might be used to improve our ability to more readily diagnose blood cancers, such as MDS.

## 2. Recent Progress on Cancer Diagnosis Using Flow Cytometry

By exploring the most recent advancements in the diagnosis of MDS and more common forms of blood cancer, such as leukemia, we aim to create a standard of biomarkers and conditions that can be used to evaluate the potential effectiveness of microflow cytometry as a diagnostic tool.

### 2.1. MDS Diagnosis

A MDS diagnosis requires a mixture of blood tests, a bone marrow biopsy, and genetic analysis. These samples are then analyzed for dysplastic morphology, genetic mutations, cytopenia, and blast percentage within peripheral blood or bone marrow biopsy [29,30].

**Figure 4:**
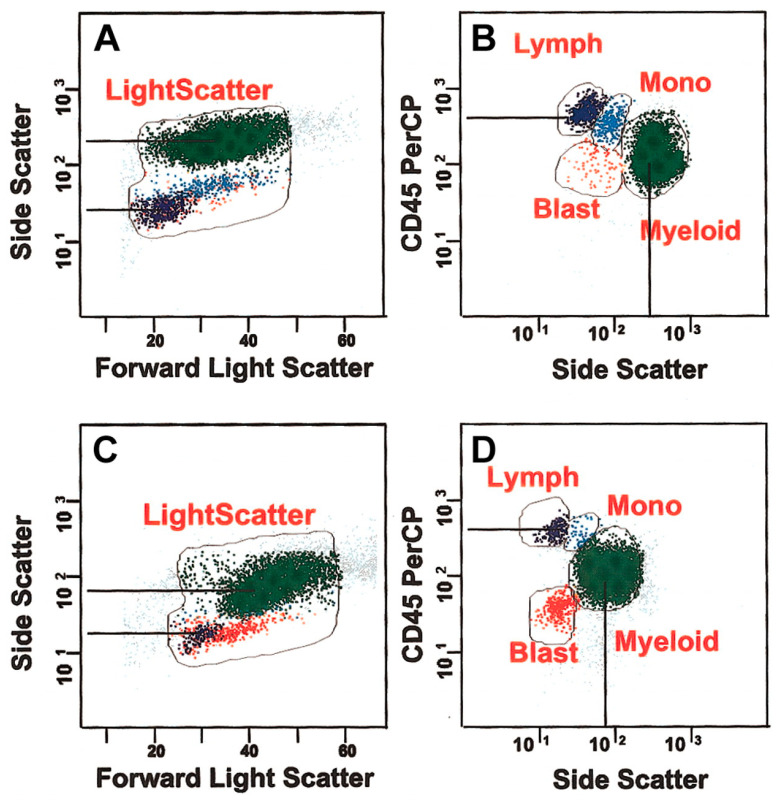
A comparison of the log difference of SSC between maturing myeloid cells and regular lymphocytes, as well as CD45 versus side scatter (**A**,**B**). Dysplastic marrow log difference between myeloid cells and lymphocytes (**C**,**D**). Results from Wells et al. [34].

Blasts are immature white blood cells found within bone marrow (BM) and are typically not found within the blood of healthy individuals. These ratios of these cells to the total BM cell population are crucially used in diagnostics for blood-related disorders and are considered to have abnormal growth if they are greater than 5% of the BM [31]. Currently, analyzing bone marrow samples for the risk of being greater than this value is an important factor in the diagnosis of flow cytometry-based MDS, and is involved as a criteria within various scoring systems for MDS, such as the IPSS-R [13,17]. Methods such as immunohistochemistry have shown some success in identifying these blasts, but they face shortcomings such as not being able to identify all blasts as some lack the unique markers for antibody tagging [32]. Flow cytometry allows for the direct analysis of these blasts, as different combinations of FLS and SSC profiles can enhance the resolution of the target molecules of interest, ultimately improving the accuracy of analyzing these blasts. Intercellular antigens for malignant neoplasms such as B cells, T cells, and erythroblasts are generally combined with reagents such as fluorochrome-conjugated antibodies against cell surface, cytoplasmic, or nuclear antigens. As these leukocytes pass through the flow cytometer, the measurement of FLS from the sample demonstrates the surface area or size of the cell, while the light refracted by the cell (SSC) is proportional to the granularity and complexity of the cell [33]. Thus, as cells pass through and are excited by different wavelengths of lasers, the fluorescent dye or fluorochrome emits light signals, which are directly proportional to the expression level of the antibody-targeted antigen. In the case of white blood cells such as blasts, identification can be performed with a flow cytometer by targeting the CD45DIM biomarker using SSC light. Therefore, in principle, blast detection and quantification of samples associated with MDS are restricted to profiles that combine the CD45DIM biomarker with a low or intermediate level of SSC light. The analysis of log differences regarding side scatter between normal lymphocytes and myeloid cells is typically nearly half a log higher than dysplastic marrow, as demonstrated within the figure [34] (Figure 4). A lower log difference and decreased granularity indicate decreased granularity, and ultimately indicate potential MDS. However, this poses one of the biggest challenges in MDS diagnosis. Many of the conventional blast-counting methods do not account for the potential for abnormal blasts to mature along the monocyte or neutrophil lines, which may reduce the number of blasts observed in this CD45/SSC region and may even provide an inaccurate number for MDS diagnosis. In addition to the potential for reduced blast counts observed in the CD45/SSC profile, the use of flow cytometry produces higher-on-average blast counts than direct counts in morphological analyses. These factors affecting the blast counts required for MDS diagnosis may initially appear to hinder the potential for flow cytometry as an effective diagnostic method. However, these limitations are overshadowed by the improved detection limit, turnaround time, and selectivity seen in flow cytometry-based tests.

A change in blood marker expression, or cytogenetics, is often observed when the patient is in a state of dysplasia (the presence of abnormal tissue preceding the formation of cancerous cells). This dysplasia is commonly uniquely identified by the absence of decreased expression of CD45 and CD117 biomarkers. Similar to how CD45/SSC profiles are used to evaluate the blast count for MDS diagnoses, the analysis of other biomarkers in tandem with a CD45/SSC profile may be used to further enhance the accuracy of the diagnosis. An important secondary biomarker for the determination of dysplasia is the increased or anomalous expression of CD34, which can therefore be used to act as a safeguard for irregular CD117 counts. It has been previously shown that some dysplastic precursors are not capable of expressing CD34 but still show decreased CD45 and CD117 counts [35]. Conversely, some patients who had shown both increased CD117 and CD34 and decreased CD34, still experienced a poor prognosis. Thus, this combination of CD45, CD117, CD34, and SSC flow cytometry analyses appears to maintain significant value for an accurate diagnostic outcome for MDS.

Despite this, it is important to note that conventional flow cytometry is typically considered an aspect of MDS diagnosis as it is used in conjunction with other techniques such as bone marrow morphology, cytogenetics, and molecular abnormalities. This is typically due to the intrinsic limitations of flow cytometry in visualizing megakaryocytes, in combination with the fact that there is a lack of markers to track megakaryocytic differentiation [36]. Other limitations associated with flow cytometry may also contribute to its role as a singular part of MDS diagnosis, such as the subjectivity of population gating strategies and the labor intensive process associated with large modern flow cytometer antibody panels [37].

### 2.2. Leukemia Diagnosis

One disease that is also heavily related to MDS is leukemia, with approximately 10–35% of MDS cases becoming acute myeloid leukemia [38]. Leukemia and myelodysplastic syndromes (MDS) are both disorders that affect the bone marrow and blood cells, sharing common biomarkers that aid in their diagnosis and classification. Leukemia is characterized by the abnormal growth of lymphoid or hematopoietic cells in the bone marrow, often affecting peripheral blood and other organs. It can be acute or chronic based on its progression rate and further specified into various subtypes according to the WHO classification, including acute lymphoblastic leukemia (ALL), acute myeloid leukemia (AML), chronic lymphocytic leukemia (CLL), and chronic myeloid leukemia (CML) [39].

Advancements in instrumentation and reagents have significantly enhanced the utility of flow cytometry as a vital tool for immunophenotyping, crucial in diagnosing various types of leukemia. It facilitates the swift identification of abnormal cell populations, characterizes their phenotype, and classifies lineage, aiding in the diagnosis or narrowing down of differential diagnoses. Additionally, flow cytometry can evaluate the clonality of mature B-cell or T-cell populations and determine DNA ploidy, aiding in diagnosis and predicting prognosis. However, accurate interpretation of flow cytometry results necessitates correlation with morphology, clinical information, and sometimes cytogenetic/molecular findings. This technique is instrumental in identifying leukemic cells in patients by analyzing specific cell biomarkers. It distinguishes between normal and abnormal cells by detecting unique combinations of biomarkers expressed by abnormal cells using fluorescently labeled antibodies. Samples are passed through a flow cytometer, where antibodies bind to target markers, causing cells to emit fluorescence. The emitted light is measured, identifying different cell populations based on their marker expression profiles. This rapid analysis of thousands of cells in a sample is essential in diagnosing and managing leukemia. However, biomarkers vary among leukemia classifications (ALL, AML, CLL, and CML), with some overlap [33].

ALL is a type of cancer that starts in the bone marrow and affects the lymphoid cells. It is characterized by the rapid and uncontrolled growth of immature lymphocytes, known as lymphoblasts. These abnormal cells crowd out normal blood cells in the bone marrow, leading to a decrease in the production of normal white blood cells, red blood cells, and platelets. In ALL, flow cytometry is used to detect specific biomarkers on the surface of leukemic lymphoblasts (immature white blood cells). These biomarkers help differentiate between normal and abnormal cells, aiding in the diagnosis and classification of ALL [40]. Common biomarkers used in the detection of ALL are described in Table 2, along with their respective effectiveness at diagnosis, such as sensitivity, specificity, positive predictive value (PPV), and negative predictive value (NPV).

CLL is a type of cancer that starts in the bone marrow and affects lymphoid cells, specifically B lymphocytes. CLL is characterized by the gradual accumulation of abnormal lymphocytes in the blood, bone marrow, and lymphoid tissues. Similar to ALL, flow cytometry is used to detect specific biomarkers on the surface of leukemic lymphocytes (mature white blood cells) in CLL [46]. Table 3 showcases the prognostic markers used for the detection of CLL, as well as their role in the prognosis of the disease.

AML is a type of cancer that starts in the bone marrow and affects the myeloid cells, which are a type of white blood cell that normally matures into red blood cells, platelets, and other types of white blood cells. In AML, the myeloid cells do not mature properly and accumulate in the bone marrow, interfering with the production of normal blood cells. AML is a rapidly progressing cancer that can occur at any age but is more common in older adults. When it comes to diagnosis, flow cytometry is used to detect specific biomarkers on the surface of leukemic myeloblasts (immature myeloid cells). These biomarkers help differentiate between normal and abnormal cells, aiding in the diagnosis and classification of AML [58]. The various types of biomarkers used within AML, along with their prognostic value, are highlighted throughout Table 4.

In CML, flow cytometry is not typically used as a primary diagnostic tool because the disease is characterized by the presence of the Philadelphia chromosome, which results from a specific genetic abnormality called the BCR-ABL fusion gene. Instead, the diagnosis of CML is usually confirmed through cytogenetic testing, such as fluorescence in situ hybridization (FISH) or polymerase chain reaction (PCR), to detect the Philadelphia chromosome or the BCR-ABL fusion gene. However, flow cytometry can still be used in CML to assess certain characteristics of the leukemic cells. For example, flow cytometry can be used to analyze the expression of surface markers on leukemic myeloid cells, which can provide additional information about the disease. Additionally, flow cytometry can be used to monitor the response to treatment in CML by tracking changes in the proportion of leukemic cells in the bone marrow or peripheral blood [69].

Flow cytometry plays a vital role in diagnosing leukemia and its subtypes by detecting specific cell surface markers on leukemic cells. For example, CD9 expression was evaluated in AML cell lines, patients with AML, and normal donors, identifying CD9 as a cell surface protein specifically expressed on AML leukemic stem cells (LSCs) but minimally on normal hematopoietic stem cells (HSCs). The biological characteristics of CD9+ cells, including their resistance to chemotherapy drugs and migration potential, were analyzed using in vitro and in vivo assays, confirming CD9 as a promising biomarker and therapeutic target for LSCs [70]. Similarly, flow cytometry was integrated into the standard examination of whole bone marrow samples after red blood cell lysis, utilizing a CD45/side scatter (SSC) gating procedure. This method showed a stronger correlation with morphological analysis of blast cells compared to conventional forward scatter (FSC)/SSC gating, improving the phenotypic determination of blast cells by discriminating between leukemic and normal cells and identifying blast cell heterogeneity based on CD45 expression levels. Additionally, this method facilitated the analysis of leukemic blasts present in low proportions and allowed for efficient discrimination between various cell lineages [71].

CD71 (transferrin receptor 1, TfR-1) has also been utilized as a marker in diagnosing acute leukemia. A study assessed CD71 expression in acute leukemia patients and found that different subtypes displayed varying levels of CD71 expression. For instance, poorly differentiated AMLs tended to have high CD71 expression on leukemic cells, while other subtypes showed lower expression levels. CD71 may not be specific for certain subtypes, but its expression levels could help understand the dynamic processes involved in leukemia’s clonal development [72]. Additionally, flow cytometry has proven valuable in characterizing hematological malignancies based on DNA content and light scatter abnormalities. A study involving patients with various hematological malignancies demonstrated flow cytometry’s utility in assessing DNA content and light scatter patterns, highlighting specific abnormalities observed across different types and grades of leukemia [73].

The aforementioned flow cytometry-based advancements have since demonstrated their value in the diagnosis of blood cancers by facilitating more rapid and accurate detection of abnormal cell population phenotypes directly linked to MDS or leukemia in over 94% of AML cases [74]. Some of the most common flow cytometry limitations to note for the diagnosis of leukemia are demonstrated through a study by Virk et al., which described the ability of flow cytometry to detect ALL in terms of minimal residual disease monitoring. This study noted that chemotherapeutic reagents can frequently cause immunophenotypic shifts within flow cytometry analysis, which may skew the interpretation of results. For instance, markers of leukemia such as CD10 demonstrate brighter expression on B cell precursors in end-of-induction samples when compared to post-consolidation samples. This slight skewing of results can weaken the application of flow cytometry to leukemia [75,76,77].

Despite this, with all factors considered, the previously mentioned diagnostic milestones successfully demonstrate the efficacy of flow cytometry as a capable diagnostic tool on a macro-scale and set the precedent for potentially improved applications of this detection method on a micro-scale. In summary, by leveraging the proven capabilities of macro-scale flow cytometry for the effective diagnosis of MDS and leukemia and integrating it with the benefits of microfluidics, a novel diagnostic platform for improved patient outcomes and a better understanding of these complex blood disorders may eventually be realized.

## 3. Microflow Cytometry as a Novel Integrated Platform for Diagnostics

The concept of microflow cytometry has seen increased usage and development as a diagnostic platform. For instance, continuous-flow microfluidics that have demonstrated their diagnostic capabilities are PCR-based microfluidic detectors [78] and cytotoxic-testing microfluidic platforms used in pre-human drug trials. Similarly, droplet microfluidics have been used in a myriad of novel diagnostic platforms, such as viral diagnostics and cyanobacterial monitoring [79]. To better comprehend the potential increase in diagnostic capabilities associated with microfluidic integration in flow cytometry, we must first explore what microflow cytometry is.

### 3.1. Microflow Cytometry as a Diagnostic Tool

Microfluidic devices take advantage of the difference in macro-scale fluid dynamics operating on a different set of physics than fluid dynamics at the micro-scale, to create controlled and high-throughput detection platforms capable of improving upon the physical limitations of conventional macro-scale detection methods [80]. The main difference is that micro-scale fluids allow for laminar flow to be achieved, which creates continuous parallel-moving sheets of fluids to readily move through channels, whereas macro-scale fluid dynamics experience the chaotic mixing of turbulent flow [81]. These non-mixing parallel fluid sheets achieved through microfluidics facilitate highly precise, controlled, and probabilistic experiments that simply cannot be achieved with larger fluid volumes. Many of these experiments include more recent examples of creating microfluidic integrated benchtop-scale flow cytometers that can achieve, at minimum, a comparable amount of specificity, selectivity, and robustness as a conventional flow cytometer [24,25,82,83]. However, there is a large difference in the modularity of microflow cytometers, as the integration of microfluidics facilitates numerous types of microfluidic-based platforms.

### 3.2. Microfluidic Subtypes and Classifications

**Figure 5:**
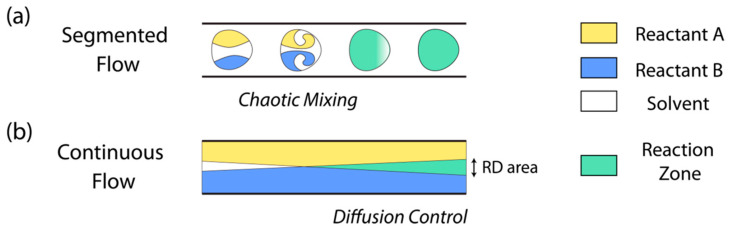
A schematic of the differences in (**a**) “segmented”/droplet-based microfluidics and (**b**) continuous-flow microfluidics. Droplet microfluidics shows the chaotic mixing occurring in each individual volume of reactant, whereas continuous flow shows a controlled and predictable mixing of fluids [82].

The two main subtypes of microfluidic devices are continuous-flow microfluidics and segmented-flow, also known as droplet-based microfluidics, both providing their own respective set of advantages and disadvantages. Some of the most important features of these subtypes are highlighted below in Figure 5.

**Figure 6:**
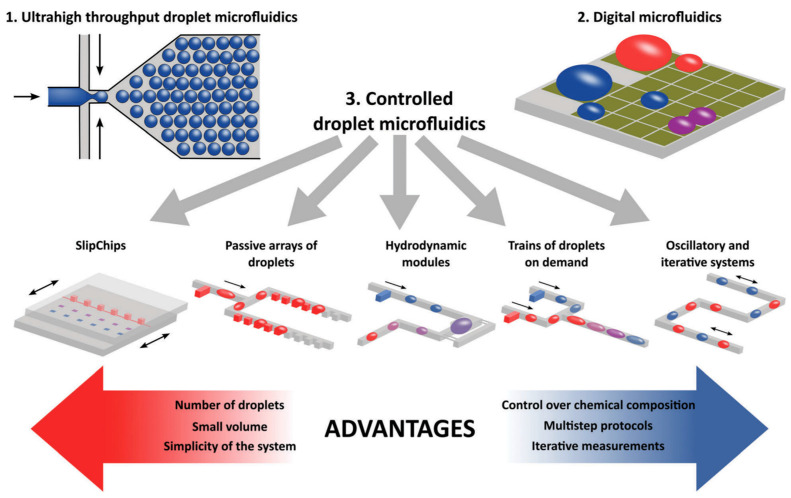
A depiction of the several different classes of droplet microfluidics. (1) High throughput microfluidics is often used to mass generate droplets purely for detection purposes; (2) digital microfluidics uses electrically conductive platforms to manipulate the behavior of droplets in real timel and (3) controlled droplet microfluidics can be used to fit the needs of the user [28].

As shown in Figure 5, the two subtypes represent the highly modular nature of microfluidic devices. While continuous-flow microfluidics are capable of highly precise and controlled fluidic dynamics capable of achieving a uniform and continuous reaction zone after the point of mixing, droplet microfluidics creates many independent sub-volumes of chaotic mixing that are carried through the rest of the device via sheath (carrier) fluid. Given their respective properties and uses, droplet microfluidics remains the favorite subtype of microfluidics between the two, as it has a greater capacity for modularity and seemingly limitless applications. Some of the most common classifications of droplet microfluidics can be seen in Figure 6 below.

As shown in Figure 6, the modularity of droplet microfluidics makes the possible applications seemingly limitless, making it a stronger candidate for its use in future diagnostic tools. Given this advantage, by integrating droplet microfluidics with the biomarkers and diagnostic research achieved in macro-scale flow cytometry, the expectation is that a microflow cytometer will provide greater diagnostic potential for blood cancers such as MDS and leukemia.

Additionally, microflow cytometry has shown great adaptability due to its ability to be integrated with various systems and adjustments for improved efficiency and further development. For instance, the integration of a microreflector with a bulk acoustic wave resonator has demonstrated greatly improved detection sensitivity within a microflow cytometer through simultaneous 3D particle focusing without the use of shear flow [84]. With microflow cytometers being able to readily be integrated within other platforms and technologies, such as spectroscopy and fluorescent microscopy, their diagnostic capabilities are extended to a wide range of biomarkers and cells.

## 4. Next Steps: MDS Diagnosis with Microflow Cytometry

Flow cytometry has gained significant traction within MDS, with current recommendations supporting the use of flow cytometry as a valuable additional diagnostic tool [15]. One of the greatest features of flow cytometry within the realm of MDS diagnosis is its ability to characterize MDS with high specificity, especially with recently developed MDS scoring criteria such as the Ogata score [85,86]. Through the assessment of various parameters in what is known as multiparametric flow cytometry, the Ogata score was developed based on four parameters: percentage of CD34+ myeloid progenitor cells among total nucleated cells; percentage of B-cell progenitors within the CD34+ subset; CD45 expression on myeloid progenitors compared to its expression on lymphocytes; and side scatter (SSC) of granulocytes compared to SSC on lymphocytes [87]. Through the validation of this score on patients with low grade MDS, this score demonstrated a specificity of 92% and a sensitivity of 69%, along with a reliable, reproducible outcome [14]. Another study in 2019 by Montauban et al. [15] analyzed 146 MDS patients, finding a sensitivity of 75.6%, a specificity of 91.2%, a positive predictive value of 95.6%, and a negative predictive value of 65.4%. It seems that the traditional Ogata score has quite moderate sensitivity in low risk MDS patients, but researchers have been investigating additional parameters and strategies that have shown to be promising in addressing this shortcoming, with a combination of the Ogata score and red score (another MDS scoring system) showing 88% sensitivity [88] and the addition of erythroid parameter evaluation showing a specificity of 95% [89].

The miniaturization of the flow cytometry process allows microflow cytometry to address many shortcomings conventional flow cytometry faces in diagnosing diseases. In a logistical sense, microchip-based flow cytometry reduces turnaround time, associated costs, and volume of reagents [90]. A study by Etcheverry et al. [91] proposed the use of silica as a material to develop a POC microchip, as it is inert, stable under pressure, and efficient at handling lasers [83]. This was due to silicon being misaligned with the device, which is pressurized, as well as auto-fluorescing at short wavelength excitation. A single cell stream was created using elastic forces and fluid inertia from the movement of cells through a viscoelastic fluid made of polymers such as polyethylene oxide (PEO) or polyvinylpyrrolidone (PVP) [91,92,93,94]. This technique allowed for a throughput of 2500 particles per second, which is significantly greater than the gold standard commercial flow cytometer’s throughput of 2000 cells per second at 20× magnification [95]. With various overlapping biomarkers between MDS and leukemia, it is plausible to say that microflow cytometry would also improve MDS throughput speed. The cost of developed microflow cytometers has also proven to be significantly lower in comparison to conventional flow cytometry, with a recent microflow cytometer costing about $60 USD and flow cytometers costing thousands of dollars. Additionally, the usage of microfluidic devices has demonstrated less need for operator intervention associated with processing samples, which can further contribute to reducing these costs [96]. With all of these factors into account, as well as the portability that such devices exhibit, it seems that microflow cytometry may be a suitable approach to POC diagnosis for MDS, a disease where early detection is beneficial due to its high lethality [10].

A study by Frankowski et al. [97] demonstrated how microflow cytometry is especially useful in differentiating hematological cells, such as leukocytes. The ability of microflow cytometry to focus sample streams restricts particle trajectories within the flow channel, which ultimately reduces surface fouling (or clumping). This study aimed to demonstrate that microflow cytometry is just as capable of blood cell identification and quantification as conventional flow cytometers. Blood samples were mixed with different solutions of fluorescently labeled antibodies, highlighting the percentage of various leukocyte subsets such as CD19+ lymphocytes and natural killer cells. The comparison of relative cell concentrations was measured between a conventional flow cytometer (MoFlo cell sorter) and a two-plate microfluidic chip made of polycarbonate. The dependence of the coefficient of variation on the sample flow rate and function of fluorescence intensity was taken into account, with a pulse height distribution of less than 2% for the chip. This is within the range of the conventional flow cytometer, which demonstrates a similar efficiency in characterizing leukocytes. With the majority of MDS score criteria being based on leukocytes such as CD71, CD117+, and various granulocytes, it seems that MDS may be a great candidate for the application of microflow cytometry [89].

Along with this groundbreaking platform, various MDS biomarkers and prognostic systems are starting to gain traction, especially in distinguishing MDS from leukemia and other hematologic disorders. A recent study by Khalilian et al. explored the relationship between TLR2, TLR4, and IRAK4 in the bone marrow of leukemia patients [98]. These values were found to be significantly different in MDS patients for diagnostic value and were found to have quite high specificity for MDS. These novel biomarkers and other parameters that meet MDS criteria, such as the WHO classification value (i.e., cytomorphology and blast count) and Ogata score [11], may be able to be quantified in a rapid, robust manner by microfluidic devices.

To summarize, microflow cytometry has shown several advantages over overflow cytometry in terms of cost, turnaround time, and operator usage. While flow cytometry has been shown to identify MDS with high specificity and sensitivity, its high cost and bulky nature make it quite inefficient for POC clinical usage. With the ability of microflow cytometers to identify and quantify most blood markers in a similar manner to conventional flow cytometry and the ability of microflow cytometry to act as a point-of-care diagnostic tool, the intersection of microflow cytometry and MDS should be further explored.

## 5. Conclusions and Future Directions

To summarize, we explored recent flow cytometry advancements within various cancers as well as their implications for MDS. Machine learning integration shows promising results in enhancing the diagnosis of lung cancers and has recently been applied to MDS. Additionally, other types of flow cytometry, such as intraoperative flow cytometry applications seem to be somewhat related to blood cancer, with the potential for simultaneous diagnoses of various conditions. With these novel concepts and advancements showing significant improvement to the diagnostic capabilities of flow cytometry, such as enhancing sensitivity and specificity and improving patient experience, it is worth further applying these concepts to MDS as well as exploring their impact on diagnosis.

The usage of microflow cytometry and its beneficial effect on leukemia diagnosis were also discussed and highlighted through the lens of MDS diagnosis. Microflow cytometry has not been explicitly explored for MDS, yet the benefits and feasibility of such a platform could potentially play a different role in the diagnosis of MDS from standard approaches such as the Ogata score. Microflow cytometry seems to be a great candidate not only for a POC MDS diagnostic platform but also for shifting the diagnosis of MDS from lab settings to on point clinical ones. While this concept has proven to bring benefits that traditional flow cytometry tends to have as a shortcoming, such as portability, operating cost, and turnaround time, there seems to be little known about how such a platform would compare in diagnostic capabilities (specificity and sensitivity) to other conventional flow cytometry-based scoring tools, most of which have recently demonstrated high specificity. In conjunction with the development of various flow cytometry and microflow cytometry advancements, the recent discovery of various biomarkers that exhibit similar morphology to these previously explored platforms even further drives the need to explore the enhancement of MDS diagnosis through these means.

There is currently a trend of recent flow cytometry advancements having several benefits over conventional flow cytometry, as well as conventional diagnostics within cancer diagnostics. Microflow cytometry also seems to follow this trend, with great potential as a POC diagnostic tool for MDS. We suggest that the application of these techniques should be further explored for MDS diagnosis in order to enhance detection, improve processing speed, and enhance accessibility to these diagnostic tools.

## Figures and Tables

**Table 1:** Common MDS scoring techniques, along with their respective criteria and effectiveness.

Scoring Technique	Criteria	Effectiveness
Ogata Score (diagnostic tool)	Score of 0 to 4, +1 for each of the following: % of CD34+ myoblasts (≥2%) % of CD34+ B-cell progenitors in CD34+ gated cells (≤5%) Ratio of mean fluorescence intensity (MFI) of CD45 on lymphocytes/MFI of CD45 on gated CD34+ myeloblasts (≤4 or ≥7.5) SSC peak channel on total granulocytic cells (CD10+ and CD10−)/SSC peak channel of lymphocytes (≤6) [14,15]	Specificty: 75.6% Sensitivity: 91.2% Positive predictive value: 92.3% Negative predictive value: 63.5% [15]
IPSS (prognosis tool)	Adds a variable number of points to score (pts) depending on the range for various variables [16] % of blast cells (0 to 2 pts, higher = more pts) Cytogenetics (0 to 1 pts depending on mutation and number of abnormalities) Number of cytopenia (0 to 0.5, higher adds more pts)	-
IPSS-R (prognosis tool)	Adds a variable number of points to score (pts) depending on the range for various variables [17]: % of blast cells (0 to 3 pts; higher = more pts) Cytogenetics (0 to 4 pts, depending on mutation and number of abnormalities) Hemoglobin concentration (0 to 1.5 pts; lower adds more pts) Platelet count (0 to 1 pts; less adds more points) Absolute neutrophil count (0 to 0.5 pts; <0.8 adds 0.5 pts)	Concordance of 0.74 for overall patient survival, concordance of 0.89 [18] for leukemia-free survival [19]
IPSS-M (prognosis tool)	Each of these factors is weighted differently based on statistical analysis. The model outputs a single number that ranks individuals in six categories, from very low to very high [20]: Hemoglobin concentration Platelet count % of blast cells IPSS-R cytogenetic risks Genetic information for 31 genes	Concordance of 0.81 for overall patient survival concordance of 0.89 for leukemia free survival [19]

**Table 2:** Biomarkers used for the detection of ALL.

Biomarker	Blood Sample	Area under the ROC Curve	Sensitivity (%)	Specificity (%)	PPV (%)	NPV (%)
miR-92a [41]	Plasma	0.755	41.5	100	100	36.7
miR-638 [41]	Plasma	0.86	54.7	100	100	42.9
TNF-α [42]	Serum	0.94	91.7	100	-	-
Survivin [42]	Serum	0.98	90	80	-	-
p53 [43]	Serum	0.8	52	100	-	-
EGFR [43]	Serum	0.93	73.9	95.8	-	-
C3f [44]	Serum	0.99	97	100	-	-
Pseudouridine [45]	Serum	-	90	97.5	-	-

**Table 3:** Biomarkers used for the detection of CLL.

Category	Prognostic Biomarkers	Prognostic Value
Serum Markers	LDT	LDT ≤ 12 months predicts a poor prognosis, LDT > 12 months correlates with a long treatment-free period and survival [47]
	s-β2M	Elevated levels predict poor outcomes and are used in risk stratification [48]
	s-TK	Elevated levels predict disease progression and are associated with shorter LDT and unmutated IGHV status [48,49]
	LDH	Indicator of time to first treatment (TTFT), associated with shorter PFS and OS [48]
Immunophenotyping	CD38	Predicts TTFT, resistance to treatment, hepatomegaly, and shorter survival [50,51]
	ZAP70	Predicts disease progression, Richter’s syndrome, and correlates with IGHV mutation status [52]
IGHV Mutation Status	Mutated-CLL and Unmutated-CLL	Unmutated status is associated with an aggressive course and predicts shorter TTFT, CD38 positivity, and resistance to treatment [53,54]
MicroRNAs	MiR-15a, MiR-16-1, MiR-34a, and MiR-155	Various impacts on prognosis in CLL, association with disease aggressiveness, and therapy response [55,56,57]

**Table 4:** Biomarkers used for the detection of AML.

Category	Prognostic Biomarkers	Prognostic Value
Genetics	FLT3 (FLT3-ITD)	FLT3 mutations in AML, particularly FLT3-ITD, are associated with poor prognosis, a higher risk of relapse, and lower overall survival rates. Screening for FLT3 mutations can help identify patients who may benefit from intensified treatment protocols or FLT3 inhibitors [59].
	NPM1	Nucleophosmin (NPM) mutations are found in 47% of AML cases with a normal karyotype, associated with a high white blood cell count and monocytic lineage involvement, but do not significantly impact complete remission or long-term outcomes, requiring further study for definitive prognostic value clarification [60].
	CEBPA	Mutations in the CEBPA gene indicate a favorable prognosis and could enhance risk assessment for AML patients with normal cytogenetics [61].
	DNMT3A	DNMT3A mutations in AML are associated with a higher risk of relapse and inferior overall survival, particularly in patients achieving complete remission. “Double-mutated” patients, with both DNMT3A and another mutation, have particularly poor outcomes [62].
	TP53	TP53 mutations in AML are independently associated with worse overall survival (OS) and disease-free survival (DFS). These mutations also correlate with specific clinicopathologic features, AML subtypes, and morphologic dysplasia [63].
	IDH 1/2	Mutations in IDH1 are associated with poor survival outcomes in a genetic subgroup that lacks FLT3 (ITD) and NPM1 (mutant). Therefore, IDH1 and IDH2 mutations are frequent in AML, and IDH1 mutations could be prognostically significant in specific AML subtypes [64].
Protomics	Calgranulin A	Analysis confirmed the expression of Calgranulin A mainly in AML patients with the worst prognosis, indicating a selective deregulation associated with poor outcomes. This suggests that the expression of Calgranulin A in leukemic cells is a predictor of low survival [65].
	UBA1, FIBA, and PF4	The peptides could serve as potential markers for monitoring minimal residual disease, assessing clinical outcomes, and predicting poor prognosis and relapse [66].
	BTG1	BTG1 might be involved in myeloid cell differentiation, suggesting its potential use as a biomarker for monitoring remission status in AML-M2 and M3 patients undergoing treatment and as a predictor of a good prognosis [67].
	Gamma 1 actin	Gamma 1 actin in AML predicts resistance to standard induction therapy, guiding the use of alternative treatments for better outcomes [68].

## Data Availability

The data presented in this study are available upon request.
